# An improved sparrow search algorithm and CNN-BiLSTM neural network for predicting sea level height

**DOI:** 10.1038/s41598-024-55266-4

**Published:** 2024-02-24

**Authors:** Xiao Li, Shijian Zhou, Fengwei Wang, Laiying Fu

**Affiliations:** 1 School of Surveying and Geoinformation Engineering, East China University of Technology, Nanchang, 330013 China; 2 Key Laboratory of Mine Environmental Monitoring and Improving Around Poyang Lake of Ministry of Natural Resources, East China University of Technology, Nanchang, 330013 China; 3 Nanchang Hangkong University, Nanchang, 330063 China; 4 State Key Laboratory of Marine Geology, Tongji University, Shanghai, 200092 China; 5 School of Geophysics and Measurement-Control Technology, East China University of Technology, Nanchang, 330013 China

**Keywords:** Ocean sciences, Physical oceanography

## Abstract

Accurate prediction of sea level height is critically important for the government in assessing sea level risk in coastal areas. However, due to the nonlinear, time-varying and highly uncertain characteristics of sea level change data, sea level prediction is challenging. To improve the accuracy of sea level prediction, this paper uses a new swarm intelligence algorithm named the sparrow search algorithm (SSA), which can imitate the foraging behavior and antipredation behavior of sparrows, to determine optimal solutions. To avoid the algorithm falling into a local optimal situation, this paper integrates the sine–cosine algorithm and the Cauchy variation strategy into the SSA to obtain an algorithm named the SCSSA. The SCSSA is used to optimize the parameter values of the CNN-BiLSTM (convolutional neural network combined with bidirectional long short-term memory neural network) model; finally, a combined neural network model (named SCSSA-CNN-BiLSTM) is proposed. In this paper, the time series data of seven tidal stations located in coastal China are used for experimental analysis. First, the SCSSA-CNN-BiLSTM model is compared with the CNN-BiLSTM model to predict the time series data of SHANWEI Station. With respect to the training and test sets of data, the SCSSA-CNN-BiLSTM model outperforms the other models on all the evaluation metrics. In addition, the remaining six tide station datasets and five neural network models, including the SCSSA-CNN-BiLSTM model, are used to further study the performance of the proposed prediction model. Four evaluation indices including the root mean squared error (RMSE), mean absolute error (MAE), mean absolute percentage error (MAPE) and coefficient of determination (R^2^) are adopted. For six stations, the RMSE, MAE, MAPE and R^2^ of SCSSA-CNN-BiLSTM model are ranged from 20.9217 ~ 27.8427 mm, 9.4770 ~ 17.8603 mm, 0.1322% ~ 0.2482% and 0.9119 ~ 0.9759, respectively. The experimental analysis results show that the SCSSA-CNN-BiLSTM model makes effective predictions at all stations, and the prediction performance is better than that of the other models. Even though the combination of SCSSA algorithm may increase the complexity of the model, indeed the proposed model is a new prediction method with good accuracy and robustness for predicting sea level change.

## Introduction

In the context of global warming, sea level rise has become a major global environmental problem, and the study of global and regional sea level changes is a hot topic in marine science at home and abroad. Direct observations of modern climate change show that the global climate system is undoubtedly warming. The sea level continues to rise due to the expansion of ocean heat and the loss of glacier material caused by global warming^1^. Global sea level rise will increase the vulnerability of low-lying coastal urban populations and ecosystems, which are often affected by natural disasters such as floods, tides and saltwater intrusion.

To actively cope with the social and economic impacts of sea level rise on coastal areas in the context of climate change, countries need the ability to make reasonable predictions of the sea level rise trends. An artificial neural network (ANN) can learn and capture trends in sea level change very effectively. As a data-driven model, an ANN can establish the relationship between inputs and outputs through repeated training. Moreover, the larger the amount of data, the better the training effect will be. Therefore, many scholars apply ANNs to sea level predictions. Röske^2^ first applied a neural network to sea level prediction for the North Sea coast of Germany, providing a new way of thinking in the sea level prediction field. Since then, neural networks have been widely used by relevant researchers in ocean predictions. For example, O. Mackarynskyy et al.^3^ have used neural networks to predict hourly sea level changes measured by tide gauges in Boat Harbor, Hillarys, Western Australia; half-day, one-day, 5-day and 10-day mean sea levels were also measured. Huang et al.^4^ developed a regional water level neural network for predicting water levels at coastal inlets, and successfully predicted water levels at local stations at coastal inlets using a series of water levels at NOAA stations at a certain distance. Karimi et al.^5^ used the adaptive neuro-fuzzy inference system (ANFIS) model, ANN model and autoregressive moving average (ARMA) model to predict and compare the sea level data series of tidal gauges in Darwin Harbor. The experimental results showed that the prediction effects of the ANFIS model and ANN model were similar and superior to that of the ARMA model. Muslim et al.^6^ used two neural networks, the ANFIS and multilayer perceptron neural network (MLP-ANN), to explore the effects of different meteorological parameters on sea level rise predictions in different periods and found that the ANFIS model had a better prediction performance than the MLP-ANN model. Guillou et al.^7^ used multiple regression methods and multilayer perceptrons to predict regional sea levels in western Brittany, France. Makarynska et al.^8^ used a feedforward neural network to predict the sea level and compared it with the actual value; the authors concluded that the method in the paper could be used for sea level prediction through evaluation indicators. Nieves et al.^9^ used Gaussian processes and recurrent neural networks to predict coastal sea level changes at regional locations on different time scales. ANNs have also been applied in storm surge forecasting^10–12^. The least squares estimation (LSE) model, multiple linear regression (MLR) model and several single neural networks were used to predict the daily mean sea level height^13^. Other scholars have used neural network models to construct ocean temperature anomaly predictions^14,15^.

All the above studies used a single neural network model to make predictions. However, every single model was not perfect and had its own limitations. With the deepening of related research, many people have combined other methods and neural networks, or a variety of neural networks, to form hybrid models for prediction. In this way, the advantages of various neural networks can be used simultaneously. Fourier transforms and wavelet transforms are the most widely used methods for denoising signal data^16^. Wang et al.^17^ proposed a method that combines wavelet decomposition and an adaptive neural fuzzy inference system (ANFIS) to construct a hybrid model capable of predicting multi-hour sea level. In 2007, researchers combined harmonics with BP neural networks to forecast tides^18^. Han et al.^19^ predicted SST by combining CNN and gated recurrent units (GRU) together with frequency analyses, and others used a network model that combines the CNN model with the long short-term memory (LSTM) model in ocean prediction^20,21^. A hybrid model can combine the advantages of several models, so the prediction ability is greatly improved.

However, whether a single model or a mixed model is used, many parameters involved in the model need to be determined by experience or trial and error, which adds subjective factors. Therefore, several researchers have turned their attention to optimization algorithms, which can be used alone or can optimize the parameters of the traditional methods and obtain more reasonable parameter settings, greatly improving the predictive performance of the model. The most widely used optimization algorithms are the genetic algorithm (GA) and particle swarm optimization (PSO). In 2004, Alvarez et al.^22^ successfully constructed a prediction model for the Ligurian Sea SST and sea level anomalies using the GA algorithm. You et al.^23^ used a GA to optimize the parameters of a two-dimensional storm surge calculation model, thereby improving the sea level prediction results. Wang et al.^24^ used a GA to optimize the parameters of a wavelet neural network for non-astronomical tide forecasting. Cheng et al.^25^ proposed an improved genetic algorithm and applied it to the optimization of reservoir systems with good results. Wang et al.^26^ proposed a hybrid genetic algorithm that combines chaos and simulated annealing methods, and the experimental results showed that the proposed hybrid algorithm is superior to both genetic algorithms and chaotic genetic algorithms. Some scholars use PSO and support vector machines to find the best value^27^. Nagappan et al.^28^ used the PSO algorithm to optimize ANN weights and predict faults in systems. Many other optimization algorithms have been applied in practice. Examples include the artificial bee colony (ABC) algorithm and ant colony optimization (ACO) algorithm^29^, the cuckoo search (CS) algorithm^30^ and the imperialist competition algorithm (ICA)^31^. Alizadeh et al.^32^ applied GA, ICA, CS and the bee algorithm (BA) to ANN training to optimize its weight and deviation values and compared them with the traditional Levenberg–Marquardt (LM) algorithm. The results show that the CS, ICA and BA algorithms are more effective than the GA and LM algorithms.

The various examples above show that a combination algorithm can improve the prediction accuracy, and the prediction performance can be further enhanced if the parameters are optimized by an optimization algorithm. Therefore, this paper combines the improved sparrow search optimization algorithm (SCSSA) with a CNN model and a BiLSTM model to propose a combination model named SCSSA-CNN-BiLSTM. The prediction ability of the model proposed in this paper is verified by using the data from multiple tide stations and comparing the results with those of four other models. The main innovations and contributions of the paper are as follows:

1. In this paper, the CNN-BiLSTM combined with a neural network model is applied to sea level time series predictions. A prediction experiment of sea level time series data from multiple tide stations shows that the combined CNN-BiLSTM model outperforms the single models in this field.
2. In this paper, a new optimization algorithm combining the sparrow search algorithm with sine–cosine and the Cauchy variation (SCSSA) is proposed and used to optimize the learning rate, the number of hidden layer nodes and the parameter values of the regularization coefficient of the CNN-BiLSTM neural network model, which avoids the unsatisfactory parameter settings caused by the artificial selection of parameter values according to experience. By comparing and analyzing the measured time series data of tide stations, it is concluded that the SCSSA-CNN-BiLSTM model is better than the CNN-BiLSTM model for sea level time series predictions.
3. In prediction analysis cases, this paper takes the measured sea level time series of six tide stations in China as the dataset and uses the single neural network models LSTM, CNN and BiLSTM and the combined models CNN-BiLSTM and SCSSA-CNN-BiLSTM for prediction and comparison. The accuracy and robustness of the SCSSA-CNN-BiLSTM model for sea level prediction are verified, and the results of this study may lead to new ideas for sea surface-related research in coastal areas.

The rest of this paper is organized as follows. The “Theory and methods” section describes the basic principles used in the experiment. The “Results” section contains predictive comparison experiments between the model proposed in this paper and the model before optimization. The proposed model and various prediction models are discussed and compared in the “Discussions” section, and finally, the concluding remarks are provided in the “Conclusions” section.

### Theory and methods

### Study area

**Figure 1:**
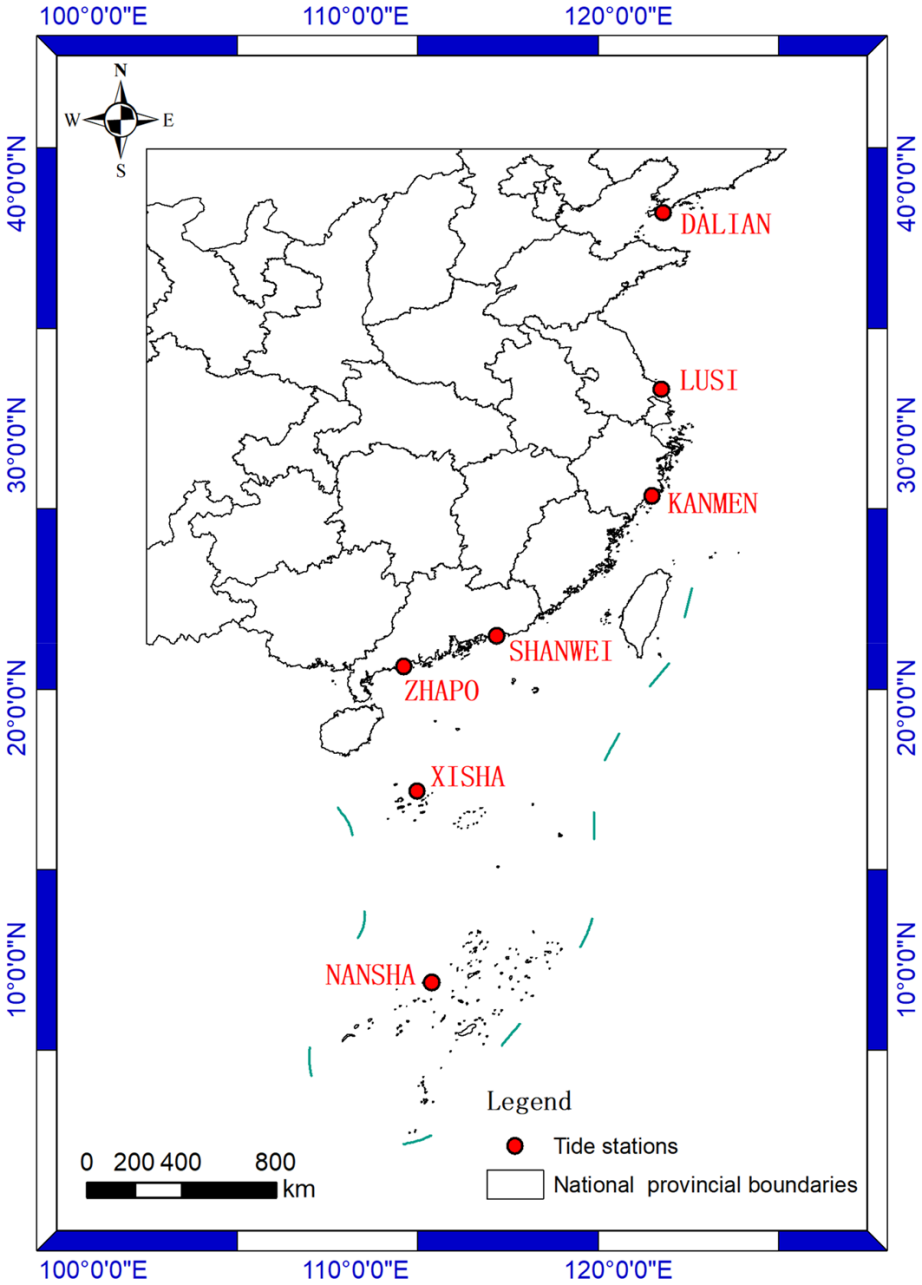
Study area map.

The study area is the coastal waters of China. To verify the reliability and applicability of the method proposed in this paper, monthly mean sea level data from 7 tide stations located in the coastal waters of China were used. The study area is shown in Fig. 1. The specific information of the data of each tide station is shown in Table 1. The missing data for each station are shown in Table 2. These missing values are supplemented by linear interpolation.

**Table 1:** Information about each tide station.

Station	ID	Country	Latitude	Longitude	Time span
SHANWEI	1406	China	22.750	115.350	1975.01–1994.12
DALIAN	723	China	38.867	121.683	1978.01–2022.12
KANMEN	934	China	28.083	121.283	1959.01–2022.12
LUSI	979	China	32.133	121.617	2000.01–2020.08
NANSHA	1730	China	9.550	112.880	2004.01–2015.12
XISHA	1745	China	16.833	112.333	1990.01–2018.12
ZHAPO	933	China	21.583	111.817	1959.01–2022.12

**Table 2:** Missing data for each station.

Station	Missing data (YYYY. MM)
SHANWEI	–
DALIAN	1979.01, 1979.02, 1996.11, 1998.09, 2000.12, 2005.03, 2006.01, 2021.03–2021.06
KANMEN	1962.09, 1993.11, 1998.09, 1999.08–1999.09, 2000.11–2000.12, 2005.03, 2006.01, 2021.03–2021.06
LUSI	2000.02, 2000.11, 2000.12, 2001.02–2001.04, 2003.08–2003.12, 2004.11–2004.12, 2005.01–2005.03, 2006.01
NANSHA	2005.03, 2006.01
XISHA	1998.09, 1999.08, 2005.03, 2006.01
ZHAPO	1998.09, 1999.08, 2005.03, 2006.01, 2021.03–2021.06

In this paper, we use China's coastal tide station’s MMSL data from the Permanent Service for Mean Sea Level (https://www.psmsl.org/data/obtaining/). The entire prediction process was completed in MATLAB 2021b software using a personal computer configured with an Intel(R) Core(TM) i5-8300H CPU, 8.00 GB of RAM, an NVIDIA GeForce GTX 1050 Ti graphics card and a Windows 10 operating system. The prediction method of this paper is single step prediction, and the lag is selected as 12. The first 12 data points of each station data in Table 1 serve as startup input variables to predict the subsequent sea level data. The first 70% of the remaining data is divided into a training set for training the model, and the last 30% is divided into a test set for testing the prediction effect of the trained model.

### Bidirectional long short-term memory neural network (BiLSTM)

**Figure 2:**
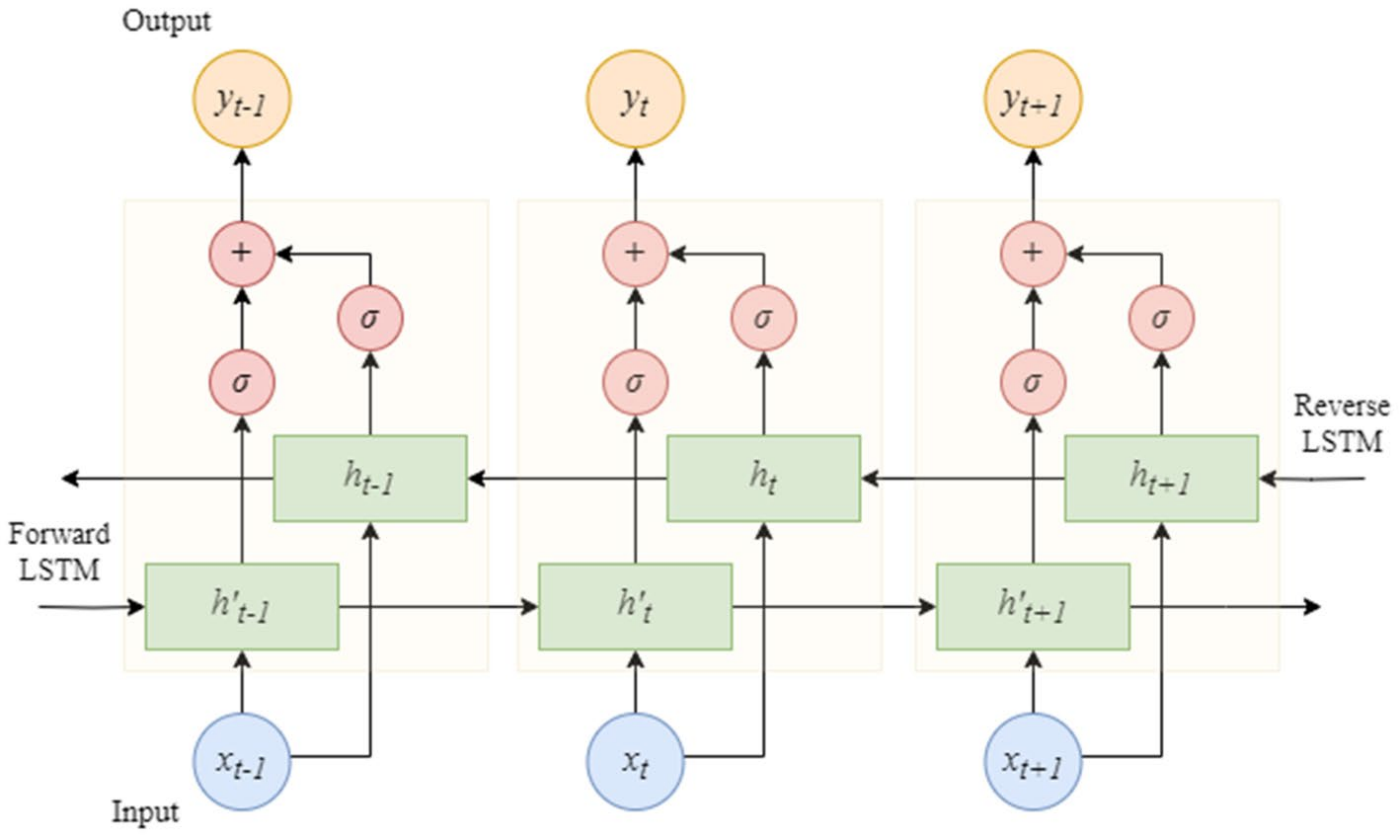
BiLSTM model structure.

The LSTM model is a variant of a recurrent neural network (RNN) in which information from each time step is no longer passed on to the next time step but rather via an additional “memory” unit. This allows LSTM to better handle long-term dependencies without the problem of disappearing gradients. LSTM introduces three gating mechanisms, namely, an input gate, forget gate and output gate, to choose to forget or retain information^33^. The BiLSTM model is further improved based on the LSTM model using a forward and reverse bidirectional LSTM so that its output results can not only use past data but can also connect with future data, which is highly suitable for processing time series data. The structure of the BiLSTM model is shown in Fig. 2, where $h_{t}$ and $h_{t}^{\prime}$ are the reverse and forward LSTM hidden layers, respectively. $x_{t}$ is the input value, $y_{t}$ is the final output value, $t$ is the $t$ th time step and $\sigma$ is the sigmoid function.

### CNN-BiLSTM

Convolutional neural network (CNN) is a class of feedforward neural networks that include convolutional computations and have deep structures. The essence of the CNN model is to build multiple convolutional filters that can extract data features and use hierarchical convolutional structures to gather input data to extract hidden topological features in the data^34^. With the increase in the number of network layers, the features extracted by the model will become increasingly abstract, and these abstract features will be integrated through the fully connected layer and then processed by the softmax or sigmoid activation function for classification or regression^35^.

**Figure 3:**
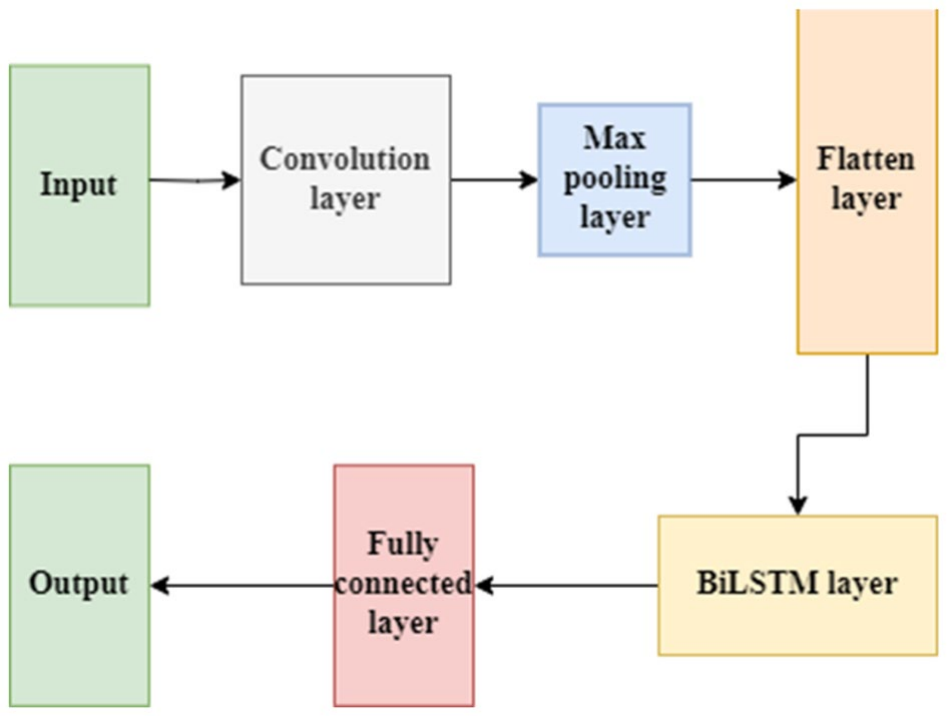
Structural diagram of the CNN-BiLSTM model.

CNN and BiLSTM are two important deep learning models. The CNN model performs well in extracting local features from the data and combining these features to form advanced features. In contrast, BiLSTM is more suitable for time expansion and has good long-term memory functions^36^. When the advantages of both methods are fully combined, the processing of the time series will improve. The structure of the CNN-BiLSTM model is shown in Fig. 3.

### A sparrow search algorithm combining sine–cosine and the Cauchy variation

The sparrow search algorithm (SSA)^37^ is an optimization algorithm proposed in 2020 that divides sparrows into three categories, discoverers, followers and observers. The discoverers can preferentially find food for the colony and guide the follower to forage. The position of sparrows can be represented in the following matrix:

1 $$X = \left[ {\begin{array}{*{20}c} {X_{1,1} } & {X_{1,2} } & \cdots & \cdots & {X_{1,d} } \\ {X_{2,1} } & {X_{2,2} } & \cdots & \cdots & {X_{2,d} } \\ {X_{3,1} } & {X_{3,2} } & \cdots & \cdots & {X_{3,d} } \\ \vdots & \vdots & \vdots & \vdots & \vdots \\ {X_{n,1} } & {X_{n,2} } & \cdots & \cdots & {X_{n,d} } \\ \end{array} } \right]$$

where $n$ is the number of sparrows and $d$ represents the dimension of the variables of the problems to be optimized. Then, the fitness value of all sparrows can be expressed by the following matrix:

2 $$F_{X} = \left[ {\begin{array}{*{20}c} {f([X_{1,1} } & {X_{1,2} } & \cdots & \cdots & {X_{1,d} ])} \\ {f([X_{2,1} } & {X_{2,2} } & \cdots & \cdots & {X_{2,d} ])} \\ {f([X_{3,1} } & {X_{3,2} } & \cdots & \cdots & {X_{3,d} ])} \\ \vdots & \vdots & \vdots & \vdots & \vdots \\ {f([X_{n,1} } & {X_{n,2} } & \cdots & \cdots & {X_{n,d} ])} \\ \end{array} } \right]$$

where $n$ is the number of sparrows , $d$ represents the dimension of the variables of the problems to be optimized, and the value of each row in $F_{X}$ represents the fitness value of the individual.

Sparrows with better fitness values have the priority to find food and become discoverers, leading the entire population to find the source of food. The discoverers' position update equation is:

3 $$X_{i,j}^{t + 1} = \left\{ {\begin{array}{*{20}l} {X_{i,j}^{t} \cdot \exp \left( {\left| {\frac{ - i}{{\alpha \cdot Iter_{\max } }}} \right|} \right),\quad R_{2} < ST} \\ {X_{i,j}^{t} + Q \cdot L,\quad R_{2} \ge ST} \\ \end{array} } \right.$$

where $X_{i,j}^{t}$ is the position of the $i{\text{th}}$ sparrow in dimension $j$ under the iteration number $t$, $\alpha \in {\text{rand}}\left( {0,1} \right]$, $Iter_{\max }$ is the maximum number of iterations, $R_{2} \in (0,1)$, it is the warning level, $ST \in \left[ {0.5,1} \right]$, represents a safe value, $Q$ is a random number that follows a normal distribution and $L$ is a matrix with a row of d-dimensional elements that are all one.

The equation for updating the followers' positions is as follows:

4 $$X_{i,j}^{t + 1} = \left\{ {\begin{array}{*{20}l} {Q \cdot \exp \left( {\frac{{X_{Worst}^{t} - X_{i,j}^{t} }}{{i^{2} }}} \right),\quad i > \frac{n}{2}} \\ {X_{p}^{t + 1} + \left| {X_{i,j}^{t} - X_{p}^{t + 1} } \right| \cdot A^{ + } \cdot L,\quad i \le \frac{n}{2}} \\ \end{array} } \right.$$

where $X_{Worst}^{t}$ is the overall worst position, $n$ is the total number of sparrows, *and *$i > n/2$ indicates that the *i*th sparrow has a poor fitness value and needs to fly to other locations to feed. $X_{p}$ is the optimal location for the discoverers, *A* is a matrix with a row of d-dimensional elements that are randomly 1 or -1 and $A^{ + } = A^{T} (AA^{T} )^{ - 1}$.

Considering the need for safe predation for the entire population, with 10% to 20% of the sparrows in the population acting as observers, the position update equation is:

5 $$X_{i,j}^{t + 1} = \left\{ {\begin{array}{*{20}l} {X_{best}^{t} + \beta \left| {X_{i,j}^{t} - X_{best}^{t} } \right|,\quad f_{i} > f_{g} } \\ {X_{i,j}^{t} + k\left( {\frac{{\left| {X_{i,j}^{t} - X_{worst}^{t} } \right|}}{{(f_{i} - f_{\omega } ) + \varepsilon }}} \right),\quad f_{i} = f_{g} } \\ \end{array} } \right.$$

where $X_{best}^{t}$ is the overall optimal position, $\beta$ is the step size correction coefficient following the normal distribution, $f_{i}$ is the fitness value of the sparrow, and $f_{\omega }$ and $f_{g}$ are the worst and best overall fitness, respectively. When $f_{i} > f_{g}$, it indicates that the sparrows are at the edge of the pack and are prone to danger; when $f_{i} = f_{g}$, it indicates that the sparrows in the pack feel the danger of the enemy and should immediately move toward the other sparrows. $k \in [-1,1]$ is a random number, and $\varepsilon$ is an extremely small constant that prevents the denominator from being zero.

During the process of hunting a sparrow, the food source may be different, as may the location. When the food found by the discoverers is locally optimal, a large number of followers will flock to the location, causing the discoverers and the entire group to stagger and lose positional diversity, thereby increasing the probability of falling into local extremes. Therefore, the sine–cosine algorithm (SCA)^38^ was introduced into the SSA in this paper, and the oscillating change characteristics of the sine and cosine models were used to determine the location of the discoverers and maintain the individual diversity of the discoverers, thereby improving the global search ability of the SSA to avoid falling into local optima.

The step search factor in the sine–cosine algorithm is as follows:

6 $$r_{1} = a - \frac{at}{{Iter_{\max } }}$$

where $a$ is a constant, $t$ is the number of iterations, and $Iter_{\max }$ is the maximum number of iterations. The step search factor shows a linear decreasing trend, which is not conducive to balancing the global search and local development capabilities of the SSA. Therefore, the step search factor is improved. The new nonlinear decreasing search factor is shown in Eq. (7). In addition, the update of the population individual position of the SSA is often affected by the current position, so a nonlinear weight factor $\omega$ is added to adjust this situation, and the mathematical equation of $\omega$ is Eq. (8).

7 $$r_{1}^{\prime} = a \times \left[ {1 - \left( {\left| {\frac{t}{{Iter_{\max } }}} \right|} \right)^{\eta } } \right]^{1/\eta }$$

where $\eta$ is the adjustment factor, $\eta \ge 1$, and $a = 1$.

8 $$\omega = \frac{{e^{{\frac{t}{{Iter_{\max } }}}} - 1}}{e - 1}$$

The new discoverers' mathematical equation then becomes:

9 $$X_{i,j}^{t + 1} = \left\{ {\begin{array}{*{20}c} {\omega \cdot X_{i,j}^{t} + r_{1}^{\prime} \cdot \sin r_{2} \cdot \left| {r_{3} \cdot X_{best} - X_{i,j}^{t} } \right|,R_{2} < ST} \\ {\omega \cdot X_{i,j}^{t} + r_{1}^{\prime} \cdot \cos r_{2} \cdot \left| {r_{3} \cdot X_{best} - X_{i,j}^{t} } \right|,R_{2} \ge ST} \\ \end{array} } \right.$$

where $r_{2} \in \left[ {0,2\pi } \right]$ and $r_{3} \in \left[ {0,2\pi } \right]$ control the movement distance of the sparrow and the influence of the optimal individual on the next position of the sparrow population, respectively.

To avoid the local optimal solution, this paper also introduces the Cauchy variation strategy into the original follower equation to obtain a new follower equation:

10 $$X_{i,j}^{t + 1} = X_{best} (t) + cauchy(0,1) \cdot X_{best} (t)$$

where $cauchy(0,1)$ is the standard Cauchy distribution function.

The Cauchy distribution is similar to the normal distribution, however, the shape of the whole distribution is flatter, and the speed of approaching the zero value is slower. Therefore, perturbation of the sparrow position update in the population with the Cauchy distribution can expand the search range of the algorithm so that it is not easy for the algorithm to fall into a local optimal situation.

After improvement, the the SCSSA algorithm procedure is as follows:

- Step 1: Initialize the population.
- Step 2: Calculate the fitness value of each sparrow to find the best and worst individuals.
- Step 3: Update the discoverer position with the new discoverers equation.
- Step 4: Update the followers’ positions with the new followers equation.
- Step 5: Update the observers’ positions using the original equation.
- Step 6: Check whether the number of iterations reaches the termination condition. If yes, go to the next step. If not, go back to Step 2.
- Step 7: The calculation is complete, and the optimal position and fitness value are displayed.

### SCSSA-CNN-BiLSTM

**Figure 4:**
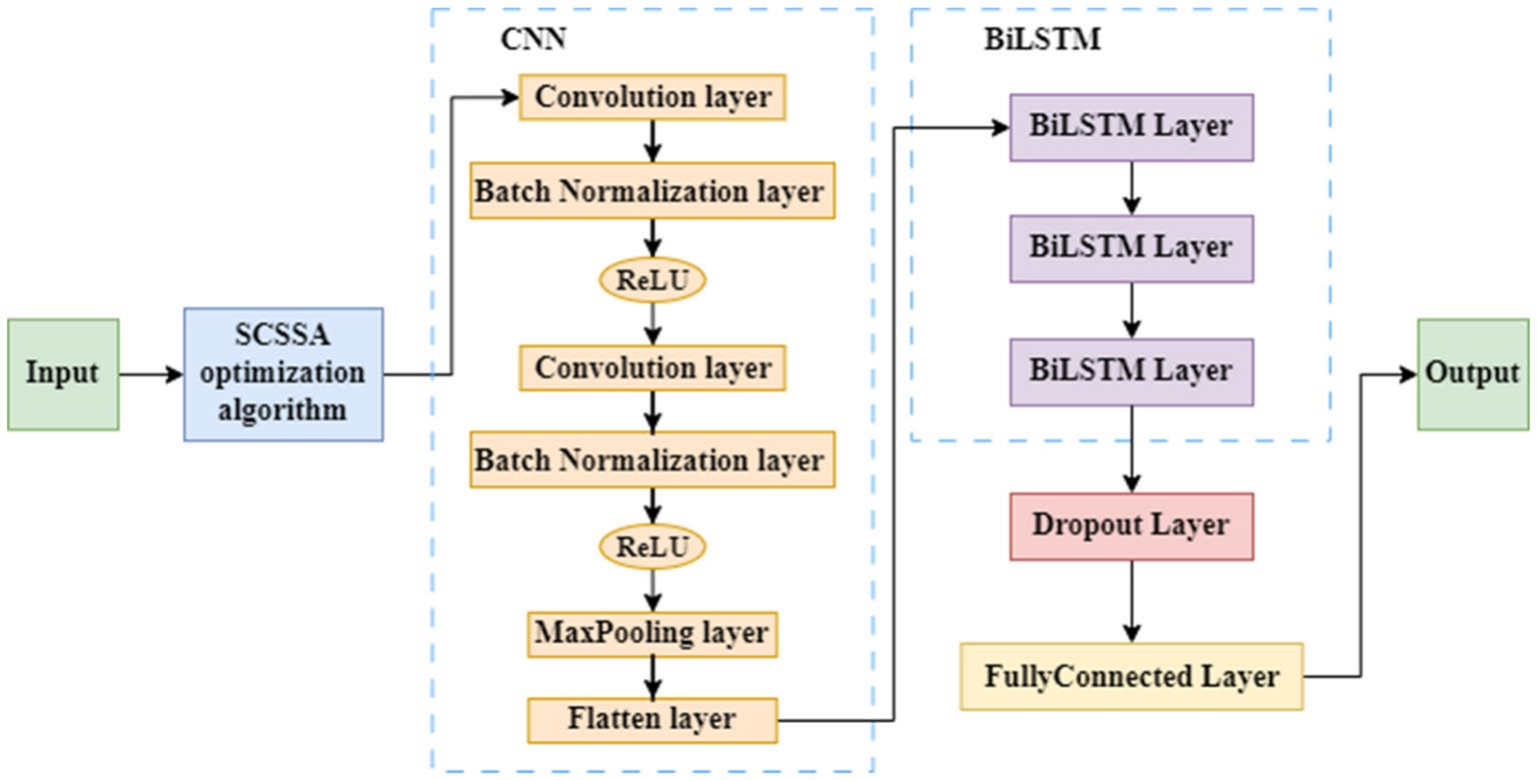
Structural diagram of the SCSSA-CNN-BiLSTM model.

**Figure 5:**
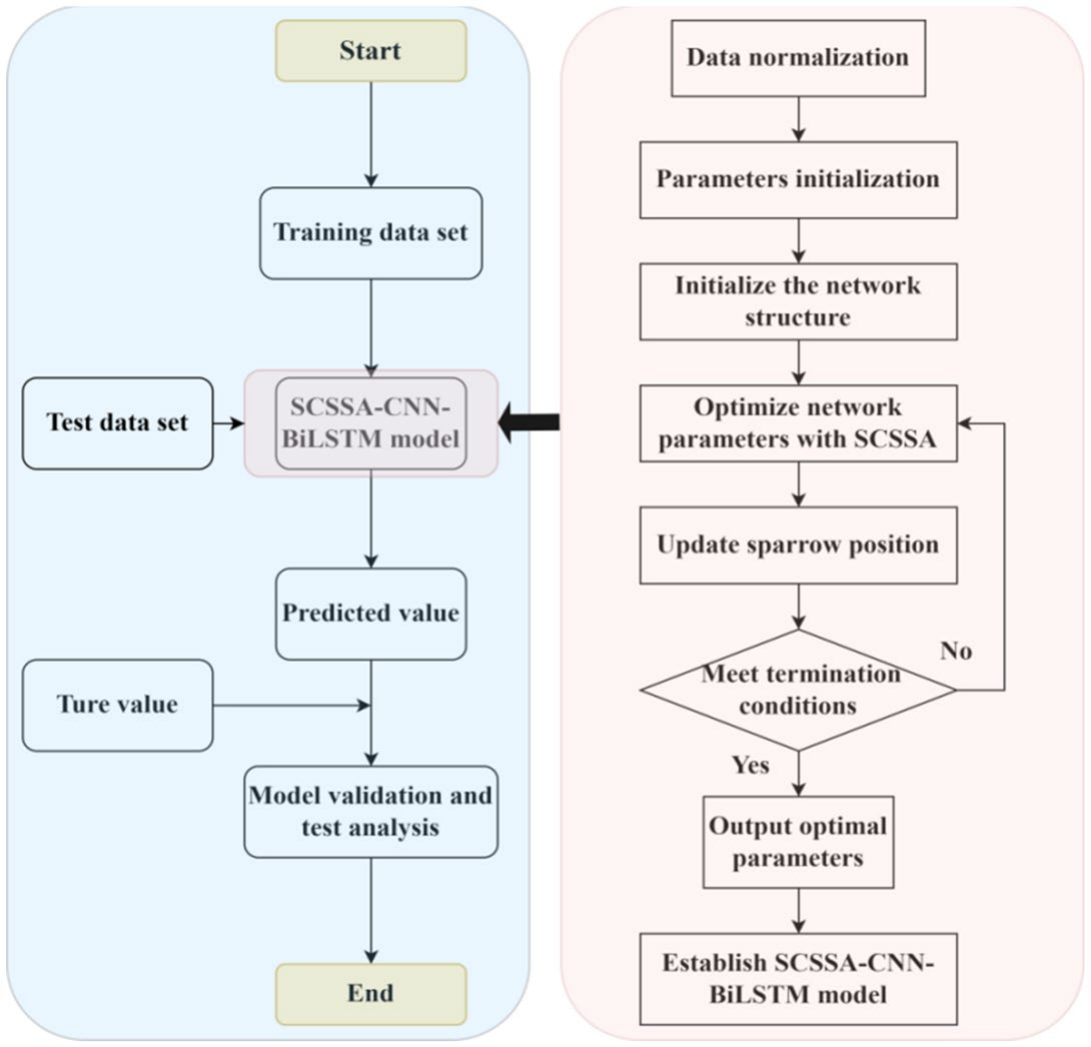
Prediction flow chart of the SCSSA-CNN-BiLSTM model.

Considering that many parameters in the CNN-BiLSTM model are manually and subjectively set, there may be unreasonable parameters. Therefore, this paper optimizes the parameters of the CNN-BiLSTM model via the SCSSA algorithm and proposes the SCSSA-CNN-BiLSTM sea-level time series prediction model. A structural diagram of the SCSSA-CNN-BiLSTM model in this paper is shown in Fig. 4. The optimization and prediction process of the entire model is shown in Fig. 5 and is divided into the following steps:

- Step 1: Divide the original data into a training set and a test set.
- Step 2: The training set is input into the model to train the model, the CNN-BiLSTM model is optimized through the SCSSA algorithm, and the optimized SCSSA-CNN-BiLSTM model is built.
- Step 3: The test set is input into the constructed SCSSA-CNN-BiLSTM model to obtain the predicted values.
- Step 4: Compare and verify the measured real values with the predicted values to evaluate the prediction effect.

### Evaluation indices

To evaluate the prediction effect of the monthly mean sea level time series of tide stations, this paper adopts four evaluation indices commonly used in the prediction field, namely, the square root error (RMSE), mean absolute error (MAE), mean absolute percentage error (MAPE) and coefficient of determination (R^2^). The equations for the four evaluation indices are as follows.

11 $$RMSE = \sqrt {\frac{1}{N}\sum\limits_{i = 1}^{N} {(x_{i} - \hat{x}_{i} )^{2} } }$$

12 $$MAE = \frac{1}{N}\sum\limits_{i = 1}^{N} {\left| {x_{i} - \hat{x}_{i} } \right|}$$

13 $$MAPE = \sum\limits_{i = 1}^{N} {\frac{1}{N}\left| {\frac{{x_{i} - \hat{x}_{i} }}{{x_{i} }}} \right|} *100\%$$

14 $$R^{2} = 1 - \frac{{\sum\nolimits_{i = 1}^{N} {\left( {\hat{x}_{i} - x_{i} } \right)^{2} } }}{{\sum\nolimits_{i = 1}^{N} {\left( {\overline{x}_{i} - x_{i} } \right)^{2} } }}$$

where $x_{i}$ is the measured value, $\hat{x}_{i}$ is the predicted value, $\overline{x}_{i}$ is the mean measurement and $N$ is the number of samples.

## Results

### Data analysis

Statistical indicators for seven stations, including the mean and standard deviation (SD), were calculated. The minimum (Min), maximum (Max) and skewness (Skew) are shown in Table 3.

**Table 3:** Statistical information for each tide station.

Station	Mean (mm)	SD (mm)	Min (mm)	Max (mm)	Skew
SHANWEI	7035	101	6824	7394	0.8204
DALIAN	7063	183	6667	7445	0.0043
KANMEN	6981	124	6709	7368	0.5544
LUSI	7074	144	6787	7387	0.1605
NANSHA	6987	78	6768	7198	0.0978
XISHA	6978	105	6772	7382	0.4999
ZHAPO	7012	124	6722	7520	0.5632

### Optimization process

**Figure 6:**
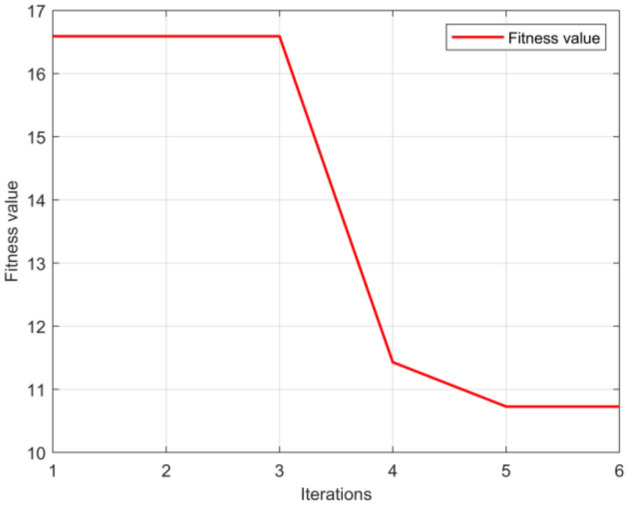
SCSSA fitness curve.

In this section, the SCSSA algorithm is used to optimize the parameters of the CNN-BiLSTM model to obtain reasonable parameter values. The CNN-BiLSTM model contains two convolutional layers and three BiLSTM layers. The training epochs of all the models were set to 300, and the initial learning rate was 0.01. The convolution kernel of the two convolutional layers is 3 times 1, the stride is 1, the activation function of the convolution layer uses the ReLU function, the pooling window size of the pooling layer is 2 times 1, and the stride is 1. The number of nodes in the BiLSTM layer is 10, both the forward and reverse LSTM gate structures in the BiLSTM layer uses sigmoid and tanh activation functions, the dropout rate is 0.2 and the regularization coefficient is set to 0.002. The initial learning rate, regularization coefficient and number of neurons in the BiLSTM hidden layer of the SCSSA-CNN-BiLSTM model are obtained by the optimization algorithm, and the other parameter settings are the same as those of the CNN-BiLSTM model. The population of the SSA is 10, the number of iterations is 6 and the selected data of the tide station are the monthly mean sea level data of the SHANWEI Station. In the optimization process, the minimum RMSE is used as the objective function. When the number of iterations was completed, each parameter value corresponding to the minimum fitness value was saved as the optimized parameter value. The fitness value curve in the optimization is shown in Fig. 6, and the range of each parameter to be optimized and the optimal parameters obtained are shown in Table 4.

**Table 4:** Values of the optimization parameters.

Parameters	Initial learning rate	Regularization coefficient	The number of BiLSTM neurons in layer 1	The number of BiLSTM neurons in layer 2	The number of BiLSTM neurons in layer 3
Range of each Parameters	0.01–0.0001	0.01–0.0001	1–300	1–300	1–300
Optimum values	0.003808	0.0001	208	27	13

### Prediction comparison

**Figure 7:**
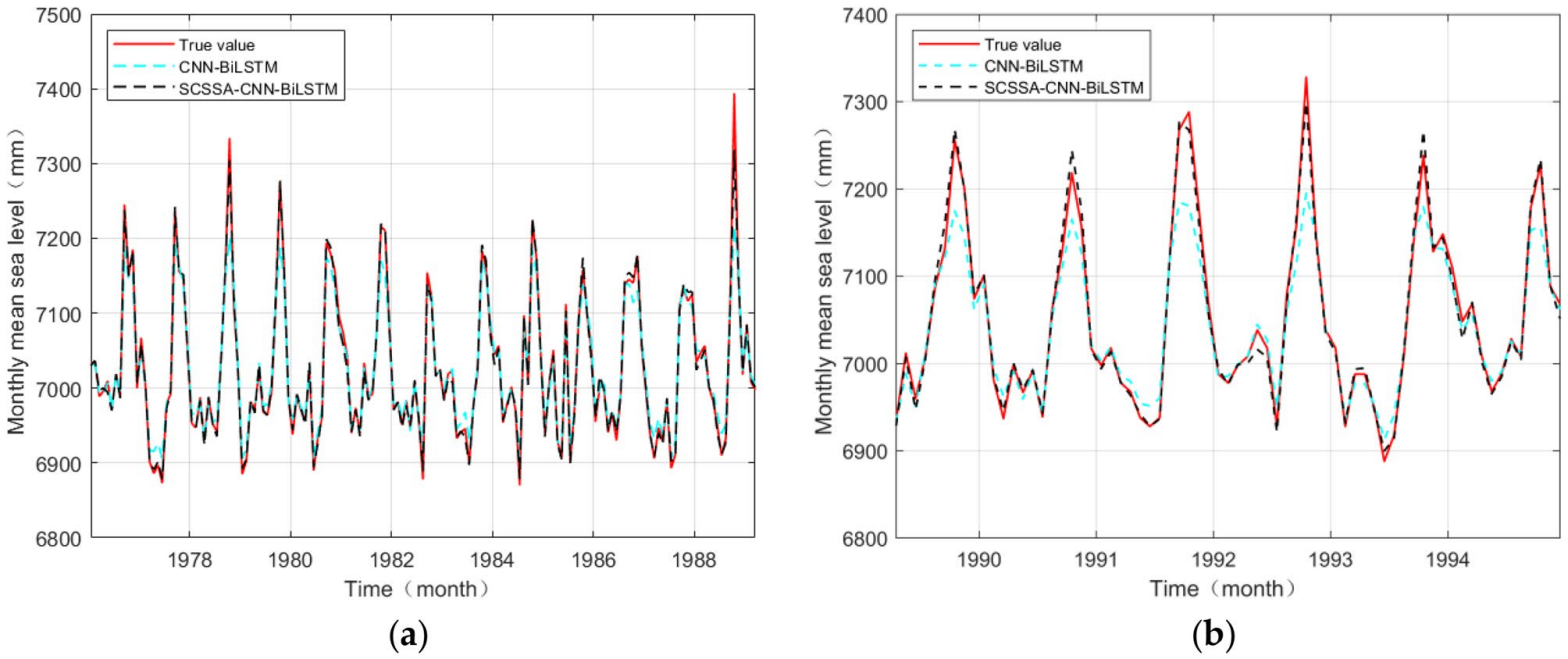
Prediction comparison between the two models at the SHANWEI station. (**a**) Training set prediction comparison and (**b**) test set prediction comparison.

To reflect the improvement of the prediction accuracy of the optimized CNN-BiLSTM model, the CNN-BiLSTM model and SCSSA-CNN-BiLSTM model were adopted to predict the monthly mean sea level data of the SHANWEI Station. The prediction diagram of the SHANWEI station data is shown in Fig. 7. As shown in the left diagram, both models better predicted the size and trend of the data in the prediction of the training set. However, the data predicted by the SCSSA-CNN-BiLSTM model are more consistent with the data of the original training set than are those predicted by the CNN-BiLSTM model. On the right, both models predict the general trend of the data, however, the data values predicted by the CNN-BiLSTM network model exhibit many obvious deviations from the original data values. The data predicted by the SCSSA-CNN-BiLSTM network model are in good agreement with the original data both in terms of value size and data trend, and the prediction effect is better.

Four evaluation indices were used to quantitatively evaluate the prediction effects of the two models, and the specific values are shown in Table 5. All the evaluation indices indicated that the SCSSA-CNN-BiLSTM model achieved the most accurate predictions, and the prediction accuracy was significantly improved by parameter optimization.

**Table 5:** Statistics of the predictive performance indicators of the two models.

	Indicator	CNN-BiLSTM	SCSSA-CNN-BiLSTM
Training set	RMSE (mm)	24.8506	9.1066
	MAE (mm)	13.9162	5.6804
	MAPE (%)	0.1963	0.0804
	R^2^	0.9349	0.9913
Test set	RMSE (mm)	32.1004	10.7267
	MAE (mm)	19.2108	7.8483
	MAPE (%)	0.2688	0.1105
	R^2^	0.8993	0.9888

### Discussions

### Comparison of prediction results of various models

In the previous section, the prediction performances of the CNN-BiLSTM model and the SCSSA-CNN-BiLSTM model were compared. To better explore the prediction ability and universality of the SCSSA-CNN-BiLSTM model, a variety of network models are used to forecast the monthly mean sea level time series of six tide stations and compare the predicted results. Considering that SCSSA-CNN-BiLSTM and CNN-BiLSTM are combinations of the CNN model and BiLSTM model, and that the BiLSTM model evolves on the basis of the LSTM model, the models selected for comparison in this section are LSTM, BiLSTM, CNN, CNN-BiLSTM and SCSSA-CNN-BiLSTM. The parameters of the various models have been obtained through multiple tests. The training epochs of all the models were set to 300, and the initial learning rate was 0.01. The LSTM model and BiLSTM model have two hidden layers, and the number of hidden layer nodes is 10. The gate structures in BiLSTM and LSTM uses sigmoid and tanh activation functions. The convolution kernel of the two convolutional layers of the CNN model is 3 times 1, the stride is 1, the activation function of the convolution layer uses the ReLU function, the pooling window size of the pooling layer is 2 times 1 and the stride is 1. The parameter settings of the CNN-BiLSTM model and the SCSSA-CNN-BiLSTM model are the same as those in the previous section.

**Figure 8:**
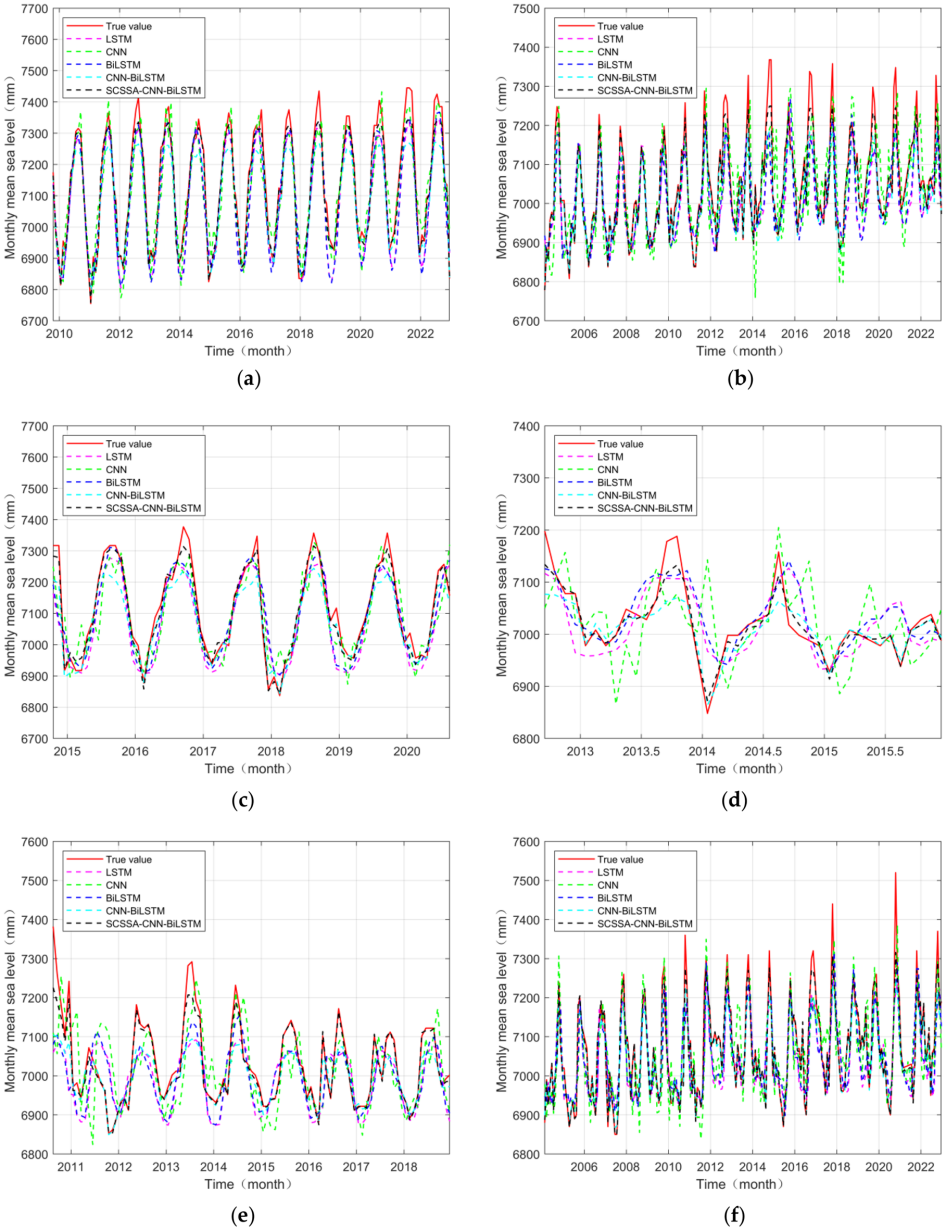
Prediction charts of various models. (**a**) DALIAN station, (**b**) KANMEN station, (**c**) LUSI station, (**d**) NANSHA station, (**e**) XISHA station and (**f**) ZHAPO station.

The test set prediction results for the data from the six tide stations are shown in Fig. 8. The figure shows that the five neural network models used at the six tide stations can all predict the test set of the data to a certain extent. Among them, the SCSSA-CNN-BiLSTM model has the best prediction effect and best fits the data of the test set; this result is more obvious for tide stations with a large amount of data. In addition, the CNN-BiLSTM model has good performance and is similar to the test set in terms of the data trend and value size. The LSTM, BiLSTM and CNN models can predict approximate data trends and values but they all have some deviations. The NANSHA, XISHA and LUSI stations have relatively large prediction deviations; the deviation is particularly prominent at the NANSHA station because this station has less data and because the model has limited data to learn during training. Due to insufficient learning, the prediction accuracy is low.

**Figure 9:**
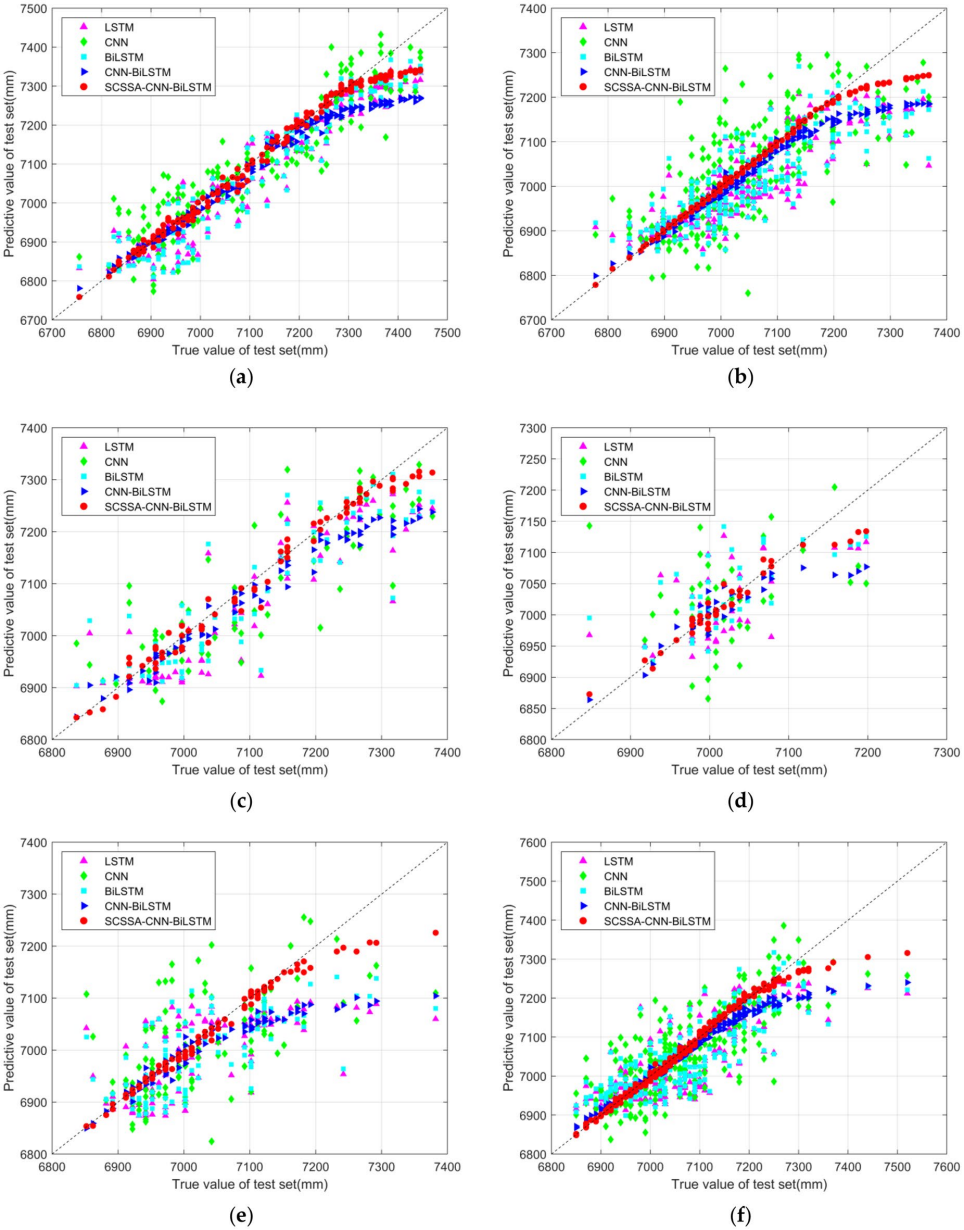
Scatter plots of the various models. (**a**) DALIAN station, (**b**) KANMEN station, (**c**) LUSI station, (**d**) NANSHA station, (**e**) XISHA station and (**f**) ZHAPO station.

As shown in Fig. 6, the scatter plot predicted by the five models on the test dataset of the six tide stations clearly reveals that most of the data points predicted by the SCSSA-CNN-BiLSTM model are concentrated near the fitting oblique line, and the best results are obtained, which are followed by those obtained by the CNN-BiLSTM model. Some values predicted by the BiLSTM model, LSTM model and CNN model differ greatly from the real values, and obvious differences are shown for the NANSHA station and XISHA station. In general, the five models are more fully trained and have more accurate prediction results at stations with more data, while the prediction results are more biased at stations with less data, which is consistent with the results shown in Fig. 9.

### Model prediction performance indicator analysis

The predicted performance indicators of the five models used are shown in Table 6. Specifically, there are relatively small gaps between the CNN model and LSTM model indicators at the DALIAN station, LUSI station, XISHA station and ZHAPO station, however, the accuracy is slightly lower at these stations. The prediction accuracy of the CNN model at the NANSHA station is relatively low. The BiLSTM model is better than the previous two indicators, however, the predicted values of these three models are biased relative to the real values. The prediction performance index of the CNN-BiLSTM model is better than that of the first three models, which shows that the prediction ability of the combined model improved substantially. The SCSSA-CNN-BiLSTM model has the strongest prediction ability among the five models; all the indicators are greatly improved based on the CNN-BiLSTM model, and the prediction error is relatively low.

**Table 6:** Statistics of the predicted performance indicators of the five models at each site.

Station	Model	RMSE (mm)	MAE (mm)	MAPE (%)	R^2^
DALIAN	LSTM	65.8084	54.2766	0.7593	0.8654
	CNN	72.6586	59.1265	0.8304	0.8359
	BiLSTM	64.2177	51.7603	0.7261	0.8719
	CNN-BiLSTM	58.6970	39.9762	0.5491	0.8930
	SCSSA-CNN-BiLSTM	27.8427	17.8603	0.2482	0.9759
KANMEN	LSTM	77.5563	60.2562	0.8486	0.5866
	CNN	90.8139	71.1800	1.0066	0.4332
	BiLSTM	75.7951	58.4640	0.8238	0.6052
	CNN-BiLSTM	43.4644	25.1598	0.3504	0.8702
	SCSSA-CNN-BiLSTM	22.4493	9.4770	0.1322	0.9654
LUSI	LSTM	77.1150	60.5206	0.8483	0.7232
	CNN	80.0454	63.6957	0.8948	0.7017
	BiLSTM	70.5125	51.8428	0.7272	0.7685
	CNN-BiLSTM	54.7238	40.2122	0.5571	0.8606
	SCSSA-CNN-BiLSTM	22.8211	17.4561	0.2451	0.9758
NANSHA	LSTM	58.2285	47.8243	0.6810	0.3251
	CNN	87.3598	69.3962	0.9893	-0.5192
	BiLSTM	55.8033	42.4020	0.6042	0.3801
	CNN-BiLSTM	39.3599	23.9795	0.3382	0.6916
	SCSSA-CNN-BiLSTM	21.0399	13.5152	0.1916	0.9119
XISHA	LSTM	89.8478	70.8346	1.0010	0.2558
	CNN	93.4580	74.2625	1.0533	0.1948
	BiLSTM	83.7694	65.4709	0.9251	0.3531
	CNN-BiLSTM	61.5742	36.6266	0.5118	0.6505
	SCSSA-CNN-BiLSTM	23.3156	11.1850	0.1574	0.9499
ZHAPO	LSTM	74.0674	57.0148	0.8088	0.6169
	CNN	79.0561	63.3405	0.8938	0.5635
	BiLSTM	72.2430	55.7241	0.7845	0.6355
	CNN-BiLSTM	39.3553	20.6215	0.2859	0.8918
	SCSSA-CNN-BiLSTM	20.9217	9.5832	0.1340	0.9694

## Conclusions

In this paper, a combined prediction model named SCSSA-CNN-BiLSTM is proposed with the use of the sparrow search algorithm, and it combines sine–cosine and a Cauchy variation to optimize the parameters of the CNN-BiLSTM model. Based on the monthly sea level time series of seven tide stations and five kinds of neural network models, a prediction experiment was constructed for a comparative analysis. By comparing the prediction performance of the SCSSA-CNN-BiLSTM model with that of the other models (LSTM, CNN, BiLSTM and CNN-BiLSTM) through a variety of comparison graphs and error evaluation indicators, the following conclusions can be drawn:

1. The quantity, regularity and stationarity of data used are crucial for neural network training, and the prediction performance maybe moderate for the stations with less available data, such as LUSI, NANSHA and XISHA stations. The predicted waveform of the DALIAN station is more consistent with the real value than that of the ZHAPO station, due to the more regular and stable data series of DALIAN station.
2. The BiLSTM model is equipped with a reverse LSTM network, and there is a forward and reverse bidirectional network, which enables the model to better understand and model the contextual information in the series, which is highly helpful for improving the time series prediction capabilities. Therefore, the performance of this model is better than that of the LSTM model. For the DALIAN station, the BiLSTM model improved the RMSE, MAE, MAPE and R^2^ by 2.42%, 4.64%, 4.37% and 0.74%, respectively, with respect to the LSTM model. Especially for tide stations with less data, the advantages of the BiLSTM model are more prominent because of the use of the reverse LSTM network; for example, at the NANSHA station, the BiLSTM model improved by 4.16%, 11.34%, 11.28% and 16.94% compared to the LSTM model on the four evaluation indices, respectively. Moreover, it is a good method for time series prediction.
3. The CNN-BiLSTM model is a combination of CNN and BiLSTM that combines the ability to extract features from the CNN model and the strong learning ability of the BiLSTM model. Based on the sea level time series prediction experiments at each tide station, the CNN-BiLSTM model yields a higher prediction accuracy and better evaluation indices. The CNN-BiLSTM model is superior to the CNN model and the BiLSTM model in terms of four evaluation indices at all the stations. Compared with those of the BiLSTM model, for the four evaluation indices of RMSE, MAE, MAPE and R^2^, the CNN-BiLSTM model can achieve maximum improvements of 45.52%, 62.99%, 63.56% and 84.23%, respectively. This shows that the CNN and BiLSTM combination model has a better prediction performance than their single models.
4. Compared with the traditional empirical method and trial and error method used to determine model parameters, the CNN-BiLSTM model, optimized by the SCSSA algorithm, can obtain more reasonable parameter values, which greatly improves the ability of the model to predict time series. With respect to the data prediction at each site, the SCSSA-CNN-BiLSTM model is far better than the other models in terms of both sequence fit and performance evaluation indicators, which effectively indicates the powerful prediction performance and high robustness of the SCSSA-CNN-BiLSTM model and provides a new way of thinking about time series prediction research.

Although the SCSSA-CNN-BiLSTM model proposed in this paper achieves excellent prediction performances in experiments, it still has limitations. For example, the combination of the SCSSA algorithm maybe more time-consuming due to the increasing model complexity. Many factors affect sea level rise, such as the sea water temperature, salinity and glacial ablation. An artificial neural network model is more effective for large-scale data and systems with complex structures; the larger the amount of data and the more types of data there are, the more accurate the prediction will be. However, in this paper, only a single time series is used for prediction, and the amount of data is small. Moreover, the article does not cover predictions of the future part of the time series; and the optimization effect of the SCSSA optimization algorithm can be compared with that of other optimization algorithms. These aspects also need further research in the future.

## Data Availability

The tide gauge datasets used in this research are freely available at https://www.psmsl.org/data/obtaining/.
